# A New Operation for Hernia*Read before the Academy of Medical and Surgical Sciences.

**Published:** 1899-06

**Authors:** Emory Lanphear

**Affiliations:** St. Louis, Mo., Fellow of the St. Louis Academy of Medical and Surgical Sciences


### A New Operation For Hernia.*

BY EMORY LANPHEAR, M. D., PH. D., ST. LOUIS, MO.,
Fellow of the St. Louis Academy of Medical and Surgical Sciences.

Having had three patients with whom complete atrophy of the testicle followed by the Bassini operation for inguinal hernia, and

*Read before the Academy of Medical and Surgical Sciences.

some recurrences when the Czerny and the Macewan methods were adopted, I some three years ago, decided to try a plan which I felt certain would prove successful, since by it total obliteration of the inguinal canal would be obtained. Experimentally the chief objection to the plan has been the reluctance of patients to accept the proposed procedure; so that thus far I have succeeded in securing but three cases. These have, thus far, been entirely satisfactory in their history subsequent to the operation.

The method is as follows: A large flap is turned back, exposing the hernial sac and the inguinal canal in their entirety. The sac is then carefully dissected out, opened and contents reduced. At this stage the opening into the abdomen is closed with gauze and the spermatic cord and testicle lifted out of their natural position, and enveloped in iodoform gauze. From the hernial sac (parietal peritoneum) there is now made a pouch, or artificial tunica vaginalis testis, into which the testicle and cord are passed and enclosed with catgut sutures in such way that not too much pressure is possible upon the cord; the whole pushed into the abdominal cavity, and anchored by a few catgut sutures. The cut in the peritoneum is next closed; next the epening into the scrotum sutured; then each muscular layer of the abdominal wall carefully sutured, completely obliterating the canal—just as is done in operating for inguinal hernia in the female.

That the ultimate fate of the buried testicle is atrophy I cannot dispute, as no opportunity has yet presented for post-mortem examination; that it is possible I cannot deny. From a surgical standpoint the chief objection to this operation is that a suppurative orchitis or epididymitis might necessitate abdominal section; but suppurative inflammation of these structures is so comparatively rare that this danger can scarcely outweigh the advantages to be gained. Thus far only the most gratifying results have been noted.

